# Supplementary material for: Identification of AURKA as a Biomarker Associated with Cuproptosis and Ferroptosis in HNSCC
Source: Int J Mol Sci. 2024 Apr 16;25(8):4372. doi: 10.3390/ijms25084372 (PMC11050640; doi:10.3390/ijms25084372)

Table S1. Top 100 cuproptosis-associated ferroptosis genes in HNSCC

cuproptosis	Ferroptosis Genes	Cor	*p* Value	Regulation
CDKN2A	CDKN2A	1	0	postive
NFE2L2	NFE2L2	1	0	postive
DLST	EIF2S1	0.817432531	2.40E-122	postive
DBT	SP1	0.70157193	6.80E-76	postive
DLAT	ATM	0.658107698	7.26E-64	postive
MTF1	MTOR	0.639692958	2.37E-59	postive
LIPT1	TUBE1	0.634959132	3.06E-58	postive
DBT	SCP2	0.602100285	4.90E-51	postive
DBT	ELAVL1	0.584570475	1.60E-47	postive
ATP7A	SETD1B	0.581901928	5.24E-47	postive
DBT	IREB2	0.581725364	5.67E-47	postive
ATP7A	SP1	0.580481826	9.83E-47	postive
ATP7A	IREB2	0.576942963	4.64E-46	postive
NFE2L2	GCLC	0.572322862	3.42E-45	postive
MTF1	SETD1B	0.572178078	3.64E-45	postive
DBT	YY1AP1	0.570538155	7.34E-45	postive
DBT	SIRT1	0.568788384	1.54E-44	postive
DBT	MTOR	0.567541288	2.62E-44	postive
PDHA1	GOT1	0.566024746	4.95E-44	postive
DBT	NRAS	0.565364775	6.53E-44	postive
DBT	PRKAA1	0.562041175	2.61E-43	postive
DBT	SETD1B	0.561490878	3.28E-43	postive
NFE2L2	PIK3CA	0.55995498	6.17E-43	postive
MTF1	MAPK14	0.559916628	6.27E-43	postive
NFE2L2	ABCC1	0.556400652	2.64E-42	postive
DBT	CS	0.553708273	7.84E-42	postive
NFE2L2	BRD4	0.550169833	3.23E-41	postive
ATP7A	LAMP2	0.54506974	2.42E-40	postive
ATP7A	SIRT1	0.543876837	3.85E-40	postive
NFE2L2	TP63	0.543726278	4.08E-40	postive
ATP7A	LPCAT3	0.541777322	8.69E-40	postive
DLAT	SLC38A1	0.540953292	1.20E-39	postive
NFE2L2	MAFG	0.538592561	2.96E-39	postive
ATP7A	ATM	0.536770414	5.93E-39	postive
NFE2L2	KLHL24	0.535610501	9.21E-39	postive
DLAT	PRKAA1	0.532677833	2.78E-38	postive
DBT	MAPK8	0.528899677	1.14E-37	postive
DBT	MAPK14	0.527287186	2.07E-37	postive
DLAT	EIF2AK4	0.524356373	6.05E-37	postive
DLAT	MTDH	0.524044758	6.78E-37	postive
ATP7A	PIK3CA	0.523929467	7.07E-37	postive
LIPT1	ISCU	0.523776011	7.48E-37	postive

ATP7A	BECN1	0.522901502	1.03E-36	postive
NFE2L2	YY1AP1	0.519899958	3.04E-36	postive
ATP7A	ABCC1	0.517185215	8.04E-36	postive
DBT	BECN1	0.515007365	1.74E-35	postive
PDHB	FANCD2	0.514674186	1.96E-35	postive
MTF1	STAT3	0.5141412	2.36E-35	postive
GCSH	PHKG2	0.512525163	4.17E-35	postive
DLD	CS	0.512339869	4.46E-35	postive
NFE2L2	KEAP1	0.512291918	4.53E-35	postive
DBT	ACVR1B	0.51055166	8.32E-35	postive
DLAT	IREB2	0.509228633	1.32E-34	postive
MTF1	BACH1	0.508937704	1.46E-34	postive
MTF1	NCOA4	0.50694671	2.90E-34	postive
MTF1	SP1	0.506823538	3.03E-34	postive
DBT	SLC38A1	0.506061936	3.94E-34	postive
DBT	ATM	0.505635352	4.56E-34	postive
NFE2L2	IDH1	0.502821114	1.19E-33	postive
DLAT	MTOR	0.499185278	4.07E-33	postive
ATP7A	BRD4	0.498983757	4.35E-33	postive
DBT	MAPK1	0.497816446	6.43E-33	postive
DLAT	ACSL4	0.496154449	1.12E-32	postive
DBT	HELLS	0.495378323	1.45E-32	postive
DBT	LPCAT3	0.492415286	3.85E-32	postive
DBT	NCOA4	0.492333854	3.95E-32	postive
ATP7A	KLHL24	0.49212242	4.24E-32	postive
DLAT	CS	0.49064745	6.87E-32	postive
PDHB	STMN1	0.490441755	7.34E-32	postive
ATP7A	STAT3	0.489476178	1.01E-31	postive
DBT	SNX4	0.489044299	1.16E-31	postive
GCSH	PRDX6	0.488586001	1.34E-31	postive
MTF1	OXSR1	0.484080343	5.72E-31	postive
ATP7A	MAPK1	0.482153166	1.06E-30	postive
LIPT1	BID	0.480397852	1.84E-30	postive
DLST	MTDH	0.47988248	2.17E-30	postive
DBT	EIF2AK4	0.479235168	2.66E-30	postive
MTF1	IREB2	0.478907884	2.94E-30	postive
DBT	ZFP69B	0.478284654	3.58E-30	postive
ATP7A	NFE2L2	0.478034824	3.87E-30	postive
DLAT	BECN1	0.477490354	4.59E-30	postive
ATP7A	NCOA4	0.476937447	5.46E-30	postive
DLD	ASNS	0.476369757	6.51E-30	postive
ATP7A	MTOR	0.472186626	2.37E-29	postive
GLS	ANO6	0.471632069	2.81E-29	postive
DBT	FANCD2	0.471026363	3.39E-29	postive

NFE2L2	ATP5MC3	0.469292343	5.75E-29	postive
DBT	STAT3	0.468317733	7.73E-29	postive
DBT	RIPK1	0.4671899	1.09E-28	postive
NFE2L2	SP1	0.466877127	1.20E-28	postive
DLAT	LPCAT3	0.466831171	1.21E-28	postive
NFE2L2	ELAVL1	0.465515097	1.80E-28	postive
ATP7A	MAFG	0.465022552	2.09E-28	postive
NFE2L2	TFRC	0.464530247	2.42E-28	postive
NFE2L2	GPX2	0.463018026	3.80E-28	postive
DLST	YWHAE	0.462827088	4.03E-28	postive
LIPT1	GLS2	0.460947243	7.04E-28	postive
DLAT	SETD1B	0.459866565	9.68E-28	postive
DBT	MAP3K5	0.459849703	9.73E-28	postive
DLAT	ANO6	0.459051522	1.23E-27	postive

Table S2. Top 10 KEGG pathway

**Descrition**	***p* Value**	**Gene ID**	**Count**
Autophagy - animal	6.9823E-23	EIF2AK4/ATG4D/ATG3/WIPI1/ATG16L1/GABARAPL1/GABARAPL2/ATG7/ATG13/ULK2/ATG5/SQSTM1/BECN1/ULK1/MAPK9/MAPK8/MAPK1/PRKAA1/PIK3CA/NRAS/LAMP2/KRAS/HRAS/HMGB1/HIF1A/MTOR/EIF2S1	27
Mitophagy - animal	5.8526E-15	GABARAPL1/GABARAPL2/ATG5/SQSTM1/BECN1/ULK1/TP53/SP1/RELA/MAPK9/MAPK8/NRAS/KRAS/HRAS/HIF1A/ATF4	16
Lipid and atherosclerosis	8.6848E-15	NOX1/XBP1/TP53/TLR4/STAT3/RELA/MAPK9/MAPK8/MAPK1/PIK3CA/NRAS/NFE2L2/NCF2/MAP3K5/KRAS/HSPA5/HRAS/CXCL2/EIF2S1/DDIT3/CYBB/MAPK14/BID/ATF4	24
Ferroptosis	1.4451E-14	SLC40A1/ATG7/LPCAT3/ATG5/NCOA4/VDAC2/TP53/TFRC/GPX4/GCLC/ACSL4/ACSL3/CYBB	13
Autophagy - other	1.4928E-14	ATG4D/ATG3/WIPI1/ATG16L1/GABARAPL1/GABARAPL2/ATG7/ATG13/ULK2/ATG5/BECN1/MTOR	12
FoxO signaling pathway	8.5487E-12	GABARAPL1/SIRT1/GABARAPL2/TGFBR1/STAT3/MAPK9/MAPK8/MAPK1/PRKAA1/PIK3CA/PCK2/NRAS/KRAS/HRAS/MAPK14/CDKN1A/ATM	17
Chemical carcinogenesis - reactive oxygen species	1.377E-11	NOX1/KEAP1/AKR1C3/VDAC2/RELA/MAPK9/MAPK8/MAPK1/PIK3CA/NRAS/NFE2L2/NCF2/MAP3K5/KRAS/HRAS/HIF1A/NQO1/AKR1C2/AKR1C1/MAPK14/ATP5MC3	21
Central carbon metabolism in cancer	2.6905E-11	GLS2/SLC7A5/TP53/SLC1A5/MAPK1/PIK3CA/NRAS/KRAS/IDH1/HRAS/HIF1A/G6PD/MTOR	13
Kaposi sarcoma-associated herpesvirus infection	7.2091E-11	ATG3/BECN1/TP53/STAT3/RELA/RB1/MAPK9/MAPK8/MAPK1/PIK3CA/NRAS/KRAS/HRAS/HIF1A/CXCL2/MTOR/MAPK14/CDKN1A/BID	19
Pancreatic cancer	8.0192E-11	TP53/TGFBR1/STAT3/RELA/RB1/MAPK9/MAPK8/MAPK1/PIK3CA/KRAS/MTOR/CDKN2A/CDKN1A	13

Figure S1. The Single immune gene infiltration characteristics of 12 cuproptosis-associated ferroptosis genes.

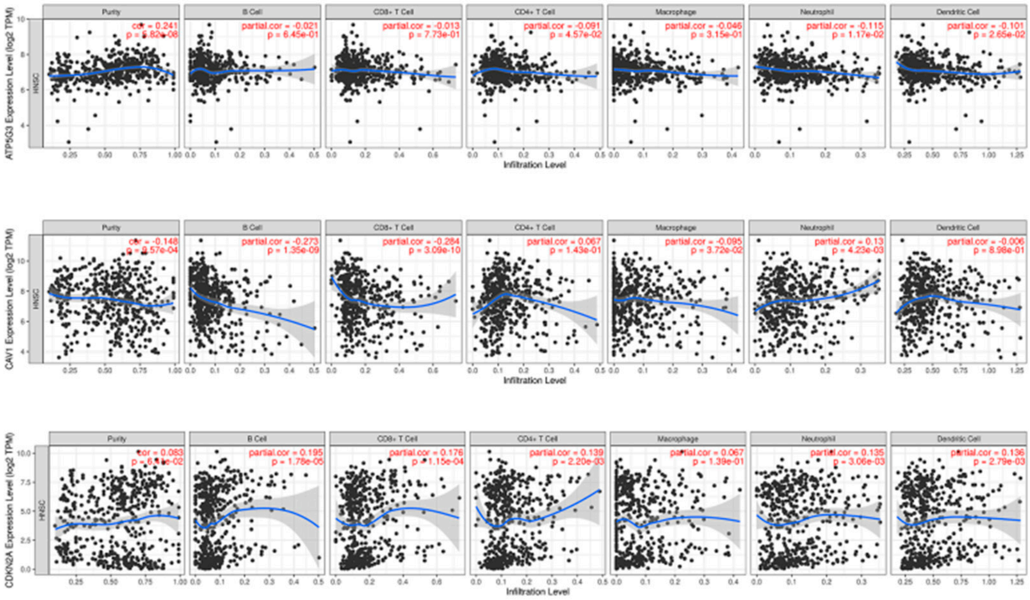

Figure S2. The Single immune gene infiltration characteristics of 12 cuproptosis-associated ferroptosis genes.

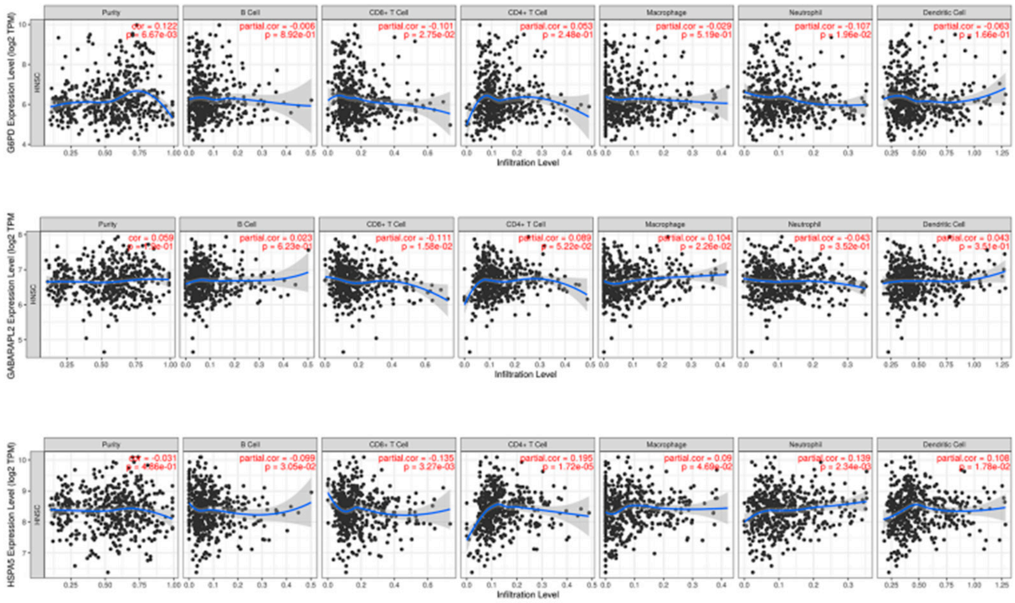

Figure S3. The Single immune gene infiltration characteristics of 12 cuproptosis-associated ferroptosis genes.

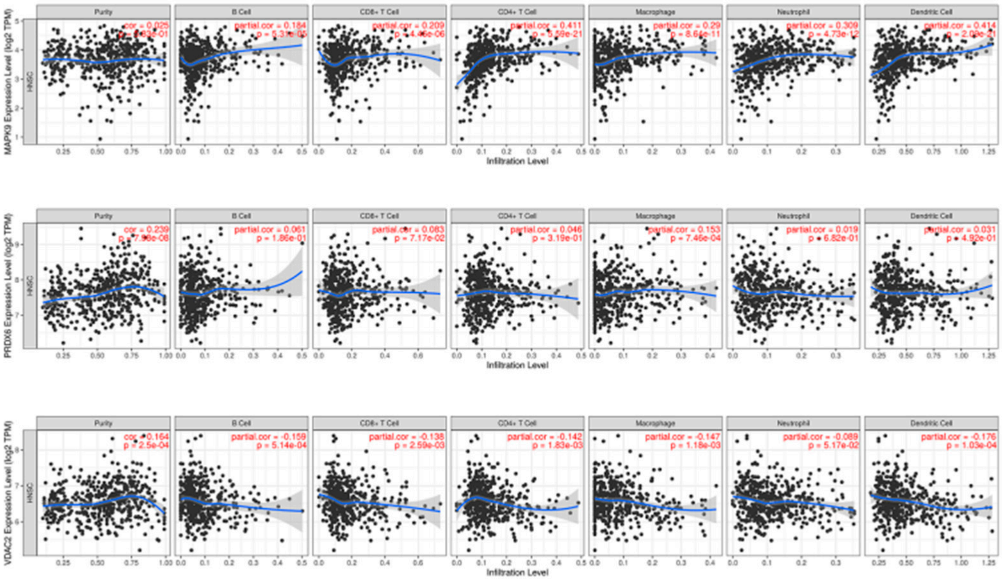

Figure S4. The Single immune gene infiltration characteristics of 12 cuproptosis-associated ferroptosis genes.

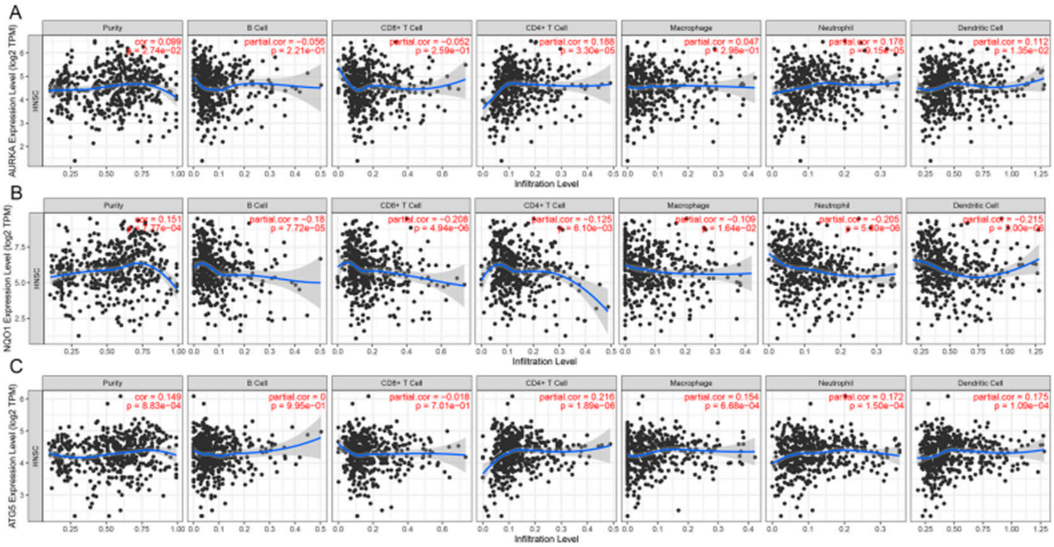

Figure S5. The pRRophetic algorithm to forecast drug sensitivity in the risk model.

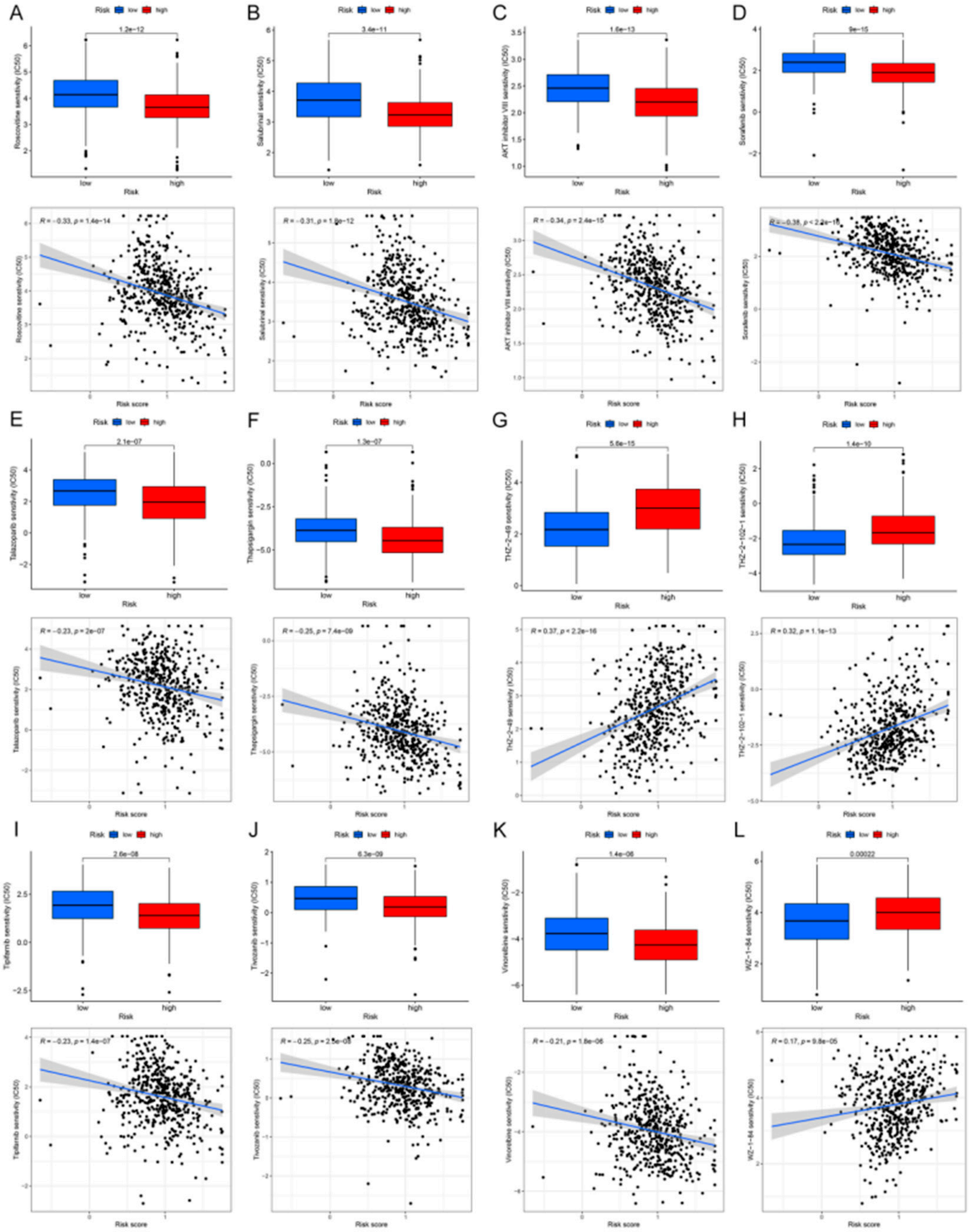

Figure S6. The RNA expression profiles of genes for which we constructed risk prognostic models.

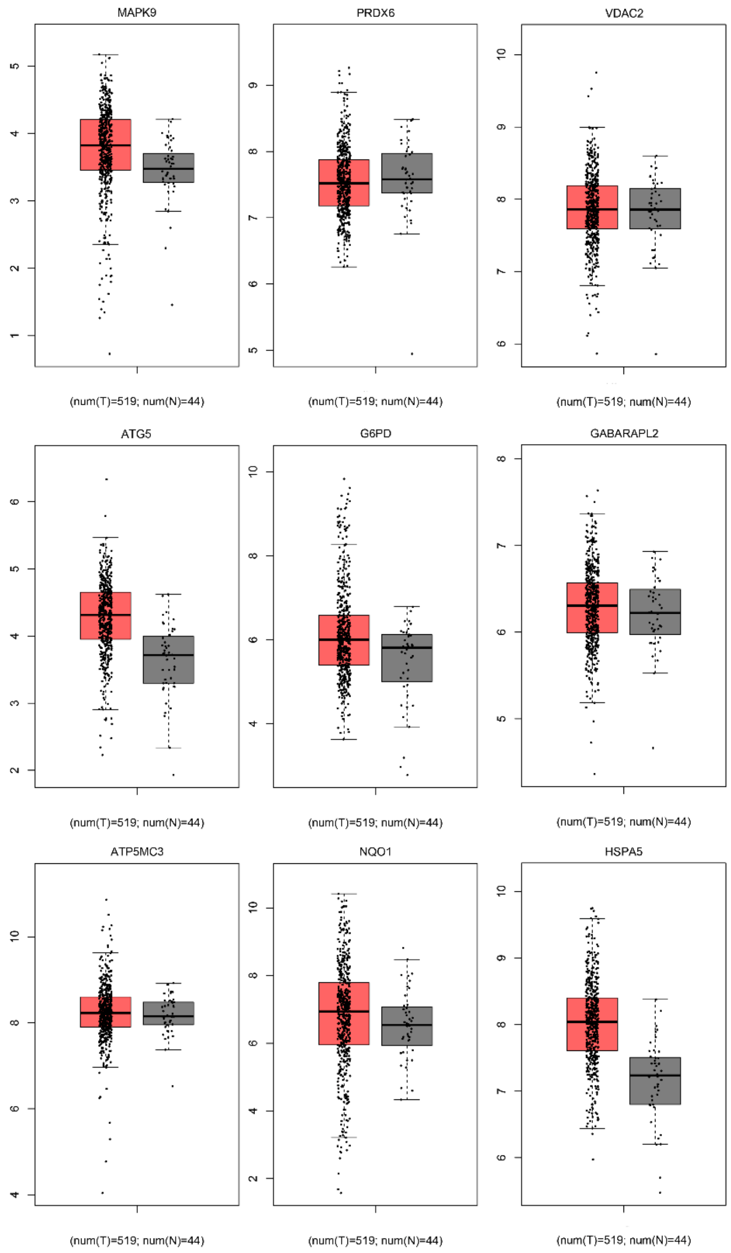

Supplement: Supplementary file 1 [file ijms-25-04372-s001.zip › ijms-2950944-supplementary.pdf]
